# *Helicobacter pylori* infection may influence prevalence and disease course in myelin oligodendrocyte glycoprotein antibody associated disorder (MOGAD) similar to MS but not AQP4-IgG associated NMOSD

**DOI:** 10.3389/fimmu.2023.1162248

**Published:** 2023-05-26

**Authors:** Chaithra Malli, Lekha Pandit, Anita D’Cunha, Akshatha Sudhir

**Affiliations:** Centre for Advanced Neurological Research, K.S Hegde Medical Academy, NIitte University, Karnataka, India

**Keywords:** *Helicobacter pylori*, environmental factor, MS, MOGAD, NMOSD

## Abstract

**Background:**

*Helicobacter pylori* (*Hp)* persists after colonizing the gut in childhood, and potentially regulates host immune system through this process. Earlier studies have shown that *Hp* infection in childhood, may protect against MS in later life. Such an association was not seen with AQP4-IgG positive NMOSD, while the association with MOGAD is unclear.

**Objective:**

To evaluate frequency of *Hp IgG* among patients with MOGAD, MS, NMOSD and matched controls and its effect on disease course. To ascertain whether childhood socio economic factors were linked to prevalence of *Hp* infection.

**Methods:**

In all, 99 patients diagnosed to have MOGAD, 99 AQP4 IgG+ NMOSD, 254MS and 243 matched controls were included. Patient demographics, diagnosis, age at disease onset, duration and the last recorded expanded disability status scale (EDSS) were obtained from our records. Socioeconomic and educational status was queried using a previously validated questionnaire. Serum *Hp*IgG was detected using ELISA kits (Vircell, Spain).

**Result:**

Frequency of *Hp* IgG was significantly lower among MOGAD (28.3% vs 44%, p-0.007) and MS (21.2% vs 44%, p-0.0001) but not AQP4-IgG+ NMOSD patients (42.4% vs 44%, p-0.78) when compared to controls. Frequency of *Hp* IgG in MOGAD & MS patients combined (MOGAD-MS) was significantly lower than those with NMOSD (23.2% vs 42.4%, p- 0.0001). Seropositive patients with MOGAD- MS were older (p-0.001. OR -1.04, 95% CI- 1.01- 1.06) and had longer disease duration (p- 0.04, OR- 1.04, 95% CI- 1.002- 1.08) at time of testing. Educational status was lower among parents/caregivers of this study cohort (p- 0.001, OR -2.34, 95% CI- 1.48-3.69) who were *Hp* IgG*+.*

**Conclusions:**

In developing countries *Hp* infection may be a significant environmental factor related to autoimmune demyelinating CNS disease. Our preliminary data suggests that *Hp* may exert a differential influence - a largely protective role for MS-MOGAD but not NMOSD and may influence disease onset and course. This differential response maybe related to immuno-pathological similarities between MOGAD and MS in contrast to NMOSD. Our study further underscores the role of *Hp* as a surrogate marker for poor gut hygiene in childhood and its association with later onset of autoimmune diseases.

## Introduction

Traditionally recognized as a human pathogen, *Helicobacter pylori (Hp)*is a gram-negative microaerophilic bacterium that colonizes the human gut in early childhood and persists most often for life (1). Infection may be acquired through oral-oral or faecal- oral transmission. Improving hygienic conditions and socio economic status has reduced *Hp* status in developed/industrialised nations. This declining trend was noted to be associated with an increase in autoimmune disorders in the developed world (2, 3) and supports the “hygiene hypothesis”. The latter refers to the inverse relationship between infection and atopy that was first proposed by Strachan (4) in 1989. He observed an increased frequency of allergic rhinitis and atopic dermatitis among first born children who were less likely to have been exposed to infection compared to younger siblings. The term “hygiene hypothesis” coined in 2000 (5), laid emphasis on the broader environmental infection burden that showed a negative correlation between overall infection frequency and a substantial increase in frequency of allergic and autoimmune diseases, observed in industrialized countries. A variety of pathogens, parasites and commensal microorganism have been observed to protect against different autoimmune conditions and includes *Hp* (6).

*Helicobacter pylori* infection in childhood possibly contributes to the development of the immune system and may be protective against later onset of some autoimmune diseases such as atopy, allergy and MS. Despite high prevalence in the developing world, < 10% develop a chronic inflammatory state which leads to symptomatic gastroduodenal disease later in the life of the human host (7). This phase of *hp* infection is also associated with extra-gastric diseases including certain autoimmune disorders such as rheumatoid arthritis, inflammatory bowel disease (8) and NMOSD (9, 10). Several studies have reported the protective role of *Hp* in MS, including from Japan (9, 10,) Iran (11), India (12) and Australia (13). There were two studies that failed to show this protective effect, one of which had included patients with opticospinal MS along with conventional MS (14). The other study had a poorly designed control arm (15, 16). Recently 2 meta analysis which included several new studies also concurred with the protective role of *hp* in MS (17, 18). As early as 2007, there were reports of a higher frequency of *Hp* among opticospinal form of MS (which in all probability was NMOSD) when compared to conventional MS patients (9, 14) This was confirmed by one of the authors (14) in a later study that incorporated AQP4-IgG assay that showed an association with NMOSD but not MS.

Myelin oligodendrocyte glycoprotein antibody associated disorders (MOGAD) have been recently discovered. Antibodies targeting MOG which is a surface expressed protein in myelin results in demyelination similar to MS (19) with which it also shares pathological similarity (20). The association of *Hp* with MOGAD has not been reported before. The present study explores the frequency of *Hp* in all three primary autoimmune demyelinating CNS disorders in a developing country where there is high *Hp* seroprevalence, to determine its possible association.

## Materials and methods

Four hundred and fifty two patients, which included all 99 myelin oligodendrocyte glycoprotein antibody associated disorder (MOGAD) (21), all 99 aquaporin-4 antibody positive (AQP4 IgG+) NMOSD (22) and 254 consecutive patients with MS (23) from the Mangalore demyelinating disease registry [MANDDIR]were selected. Two hundred and forty three healthy volunteers matched by age and gender were included as controls for this study. Patient demographics, diagnosis, age at disease onset, duration and the last recorded expanded disability status scale (EDSS) was obtained from our data base. Socioeconomic and educational status (24) was queried using a previously validated questionnaire (12). Testing for serum *Hp* IgG was done using Vircell (Granada, Spain) ELISA kits as per manufacturer’s instructions. Antibody index was determined (by dividing optical density values of samples by optical density for cut-off control samples, multiplied by 10). Antibody index was positive if >11, equivocal if between 9 -11 and negative if < 9. All equivocal results were retested and if found to remain equivocal reported as negative for *Hp* IgG. All patients were tested for both AQP4 IgG (25) and MOG IgG using “in house” cell based assays. This study was approved by the institutional ethics committee and patients and healthy volunteers signed an informed consent form.

### Statistics

Categorical variables were expressed in percentages and continuous variables as mean and standard deviations. Patients and controls were stratified by *Hp*serology. Frequency of *Hp* in different subtypes of demyelinating disorders were compared with matched controls and then amongst each sub group using Chisquare test. Univariate analysis was performed on following variables namely age at onset,disease duration, gender, EDSS, socio economic and educational state and area of living.Independent variables that showed a p value of ≤ 0.20 were included in the multivariate analysis. A p value < 0.05 was taken to be significant. Strength of association was expressed as odds ratios (OR) and 95%confidence intervals (CI). Analysis was performed on SPSS statistical software program (IBM, USA).

## Results

Clinical and Demographic features are listed in **Table 1**. Patients in the MS group were predominantly of the relapsing remitting (RR). The non MS group comprised of equal number of AQP4IgG + NMOSD and MOGAD patients. Patients were predominantly female among MS and NMOSD patients. All patients had comparable age and disease duration at time of *Hp* serology testing.

**Table 1:** Clinical and demographic features.

	MS (254)			Non MS disorders (198)		Healthy control
	RRMS (174)	SPMS (73)	PPMS (7)	AQP4-IgG+ (99)	MOGAD (99)	243
Gender (Female)	126(72.4%)	44(60.3%%)	5(71.4%)	90(90.9%)	44(44.4%)	149(61.3%)
Age (Mean ± SD)	35.18 ± 10.9	45.3 ± 11.36	45.7 ± 10.0	41.6 ± 12.6	33.06 ± 14.5	35.2 ± 11.23
Disease Duration (Mean ± SD)	8.58 ± 5.63	13.2 ± 6.13	11.7 ± 10.54	10.9 ± 7.2	6.81 ± 5.84	–
EDSS (Mean ± SD)	1.46 ± 0.97	5.41 ± 2.42	6.0 ± 3.0	4.27 ± 3.28	1.68 ± 2.02	–
*Hp*IgG +	35 (64.8%)	16(29.6%)	3(5.6%)	42 (42.4%)	28(28.3%)	107(44.03%)

HpIgG +, Helicobacter pylori antibody positive; EDSS, Expanded disability status scale; RRMS, relapsing remitting multiple sclerosis; SPMS, secondary progressive MS; PPMS, primary progressive MS, AQP4-IgG += Aquaporin-4 antibody positive, MOGAD= myelin oligodendrocyte glycoprotein antibody associated disease.

### Frequency of Hp IgG serology

*Helicobacter pylori* antibody frequency was significantly low in MOGAD (28.3% vs 44% p- 0.007) and MS patients (21.2% vs 44%, p- 0.0001) when compared to matched controls. Comparison of NMOSD patients did not show a difference from controls (42.4% vs 44% p=0.78).Seroprevalence of *Hp* was similar between MS and MOGAD (p- 0.16) while it was significantly different for both subtypes when compared to NMOSD (**Supplementary Table 1**).

### Hp serological prevalence and disease association

For determining association with potential disease modifying variables, MOGAD and MS patients were combined together (MOGAD-MS) and analysed after stratification based on *Hp* serology. In univariate analysis (**Supplementary Table 2**), age at disease onset was significantly higher in seropositive patients (p- 0.001, OR- 1.03, 95% CI -1.01-1.05) who were also less educated (p-0.009, OR-1.99, 95% CI -1.18- 3.36). In multivariate analysis- age at disease onset (p-0.001, OR-1.04, 95% CI- 1.01-1.06), duration of disease (p-0.04, OR-1.04, 95%CI- 1.002- 1.08) and educational status (p-0.02, OR- 2.12, 95% CI- 1.11-4.06) were significant association after controlling for gender, disability measured by EDSS, socioeconomic status and area of living (**Supplementary Table 3**). Among NMOSD patients, seropositive patients showed a trend for greater disability (p- 0.08, OR-1.14, 95%CI – 0.97-1.33) compared to seronegative patients. Lower socioeconomic status was significantly associated with *Hp* seropositivity (p=0.01, OR-3.83, 95% CI – 1.31 -11.23). Age of disease onset did not differ between the groups. Though statistically insignificant, our data suggested that seropositive patients may have had an early disease onset. Multivariate analysis did not show any variable to be significant (data not shown).

### Hp serology and association with socioeconomic and educational status

We combined all patients and controls, to determine whether there were commonalities among those harbouring *Hp* in our study cohort. In univariate analysis a significant number of seropositive patients had low educational levels(p- 0.001, OR-2.3, 95% CI 1.57- 3.38) and lived in rural areas(p- 0.002, OR 1.79, 95% CI – 1.23- 2.61). After adjusting for other variables, (**Tables 2**, **3**) multivariate analysis showed that educational background of care givers and patients (p- 0.001, OR -2.34, 95% CI- 1.48-3.69)was a significant determinant that differentiated seropositive patients and controls from their seronegative counterparts.

**Table 2:** Association of socioeconomic status and *hpylori* status among the study cohort.

	*Hp+*	*Hp -*	p value	Odds ratio	95%CI
Gender					
Male	77(33.3%)	159(34.3%)	0.801	0.95	0.68-1.34
Female	154(66.6%)	305(65.7%)			
Education					
Basic education	115(68.8%)	197(51%)	**0.001**	2.3	1.57-3.38
Graduation and above	52(31.2%)	189(49%)			
Socioeconomic status					
Low	81(46.8%)	167(42.6%)	0.35	1.18	0.82-1.69
High	92(53.2%)	225(57.4%)			
Area of living					
Rural	109(64.5%)	192(50.3%)	**0.002**	1.79	1.23-2.61
Urban	60(35.5%)	190(49.7%)			

(Univariate analysis for all cases and controls combined). Bold value indicates a significant p value of < 0.05.

**Table 3:** Association of socioeconomic status and *hpylori* status among the study cohort.

Variables	p value	Odds ratio	95%CI
Education status	**0.001**	2.34	1.48-3.69
Area of living	0.14	1.38	0.89-2.13
Socioeconomic status	0.37	0.82	0.54-1.25

(Multivariate analysis for all case and control combined). Bold value indicates a significant p value of < 0.05.

## Discussion

*Helicobacter pylori* prevalence remains high in developing nations and offers an opportunity to study its association with autoimmune demyelinating CNS disorders in these regions. This is particularly relevant for Indian MS patients in whom there were no definitive environmental associations detected that were traditionally associated among white populations. These include Epstein Barr virus infection (26), smoking (12), obesity (12) and Vitamin D deficiency (27). In an earlier study we reported that *Hp* infection was protective against MS patients (12) and we have currently replicated the results in a larger cohort. Seropositive patients with MS- MOGAD had a later age of disease onset in our study. A similar delay in disease onset was reported in *Hp* seropositive patients with bronchial asthma (28). Despite a longer duration of disease, seropositive patients had disability that was comparable with seronegative patients (**Supplementary Table 2**). Previously two case control studies have reported similar results in *Hp* seropositive MS patients (9, 11). Our study also suggests that *Hp* infection may confer a protective effect against MOGAD similar to MS. In contrast, *Hp* infection was detected to be frequent in NMOSD patients. An effect on disease onset and course could not be clearly determined in the latter.

The differential association of CNS autoimmune disorders with *Hp* infection may in part be due to the differential immune response mounted by *Hp* in the human host (29). It is evident from human gut mucosal studies that *Hp* behaves as a commensal in early infection with a potential for pathogenicity in later life. Compared to adults, gastric mucosa in paediatric patients showed minimal inflammation, increased T regulatory (reg) cells and elevated levels of Treg related cytokines namely transforming growth factor- beta (TGFβ) and interleukin 10 (IL10) (30). Limited studies in experimental allergic encephalitis (EAE) models demonstrated that prior infection with *Hp* induced Foxp3^+^Treg cell upregulation (31) and inhibition of MOG specific Th1 and Th17 responses (32). In adult patients, peptic ulceration was noted to be associated with reduced Tregs (33).

**Figure 1:**
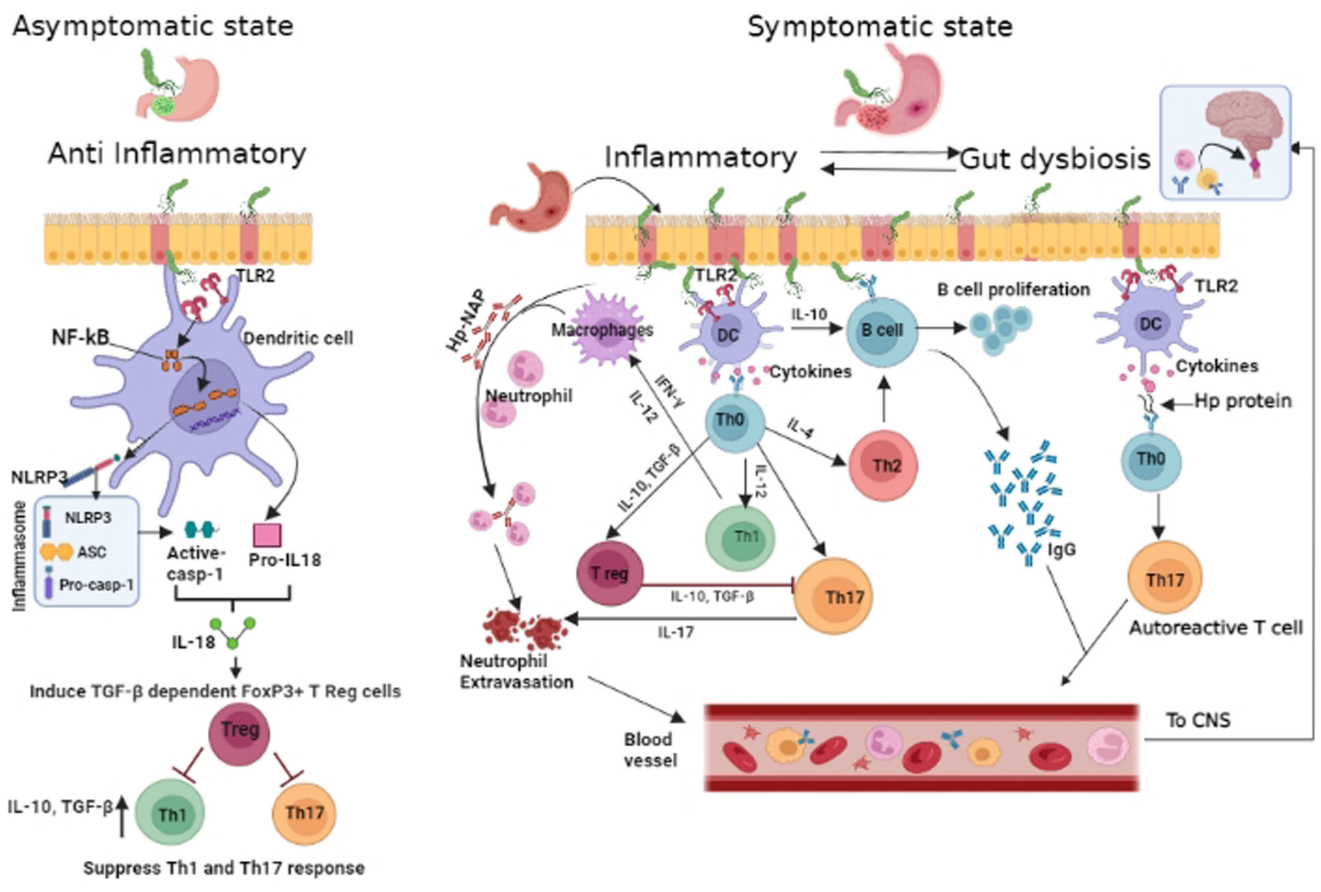
Proposed schematic of *Helicobacter pylori* mediated modulation of host immunity: In early phase of *Hp* infection(depicted on left of cartoon), *Hp*triggers Nucleotide-Binding Domain, Leucine-Rich–Containing Family, Pyrin Domain–Containing-3(NLRP3)inflammasome and caspace 1 activity in antigen presenting cell([APC]dendritic cells and macrophages) *via* Toll like receptor2 (TLR2) and Nuclear factor kappa B(NF-kB). Processing of pro interleukin -18 (pro-IL-18) and release of mature IL-18 results in T regulatory (Treg) cell differentiation and production of anti inflammatory cytokines – Interleukin-10 (IL10) and transforming growth factor Beta (TGF-β) with further expansion of Treg cells, resulting in immunosuppression. In the inflammatory state (depicted on right),APCafter processing *Hp*,signals naïve Tcells to differentiate (through release of cytokines - IL12 &IL17) into T helper (Th1 &Th17) cells andB cells are driven by interleukin 10 (IL10)to generate *H*p-specific antibodies. Th17 induced neutrophil recruitment is further enhanced by *Hp*– neutrophil activating protein (NAP). Gut derived antibodies with potential for cross reaction with neural elements, *Hp* specific autoreactive T cells, neutrophils and *Hp* specific antibodies may target the central nervous system and induce/aggravate CNS inflammation. Created in BioRender.com.

We have hypothesised possible mechanisms by which *Hp* infection could modulate the host immune response. Recent insights (34) suggest that *Hp* may possess inflammasone ligands that trigger caspase- 1 activation in dendritic cells lining the gastric lumen (**Figure 1**). Activation of the Toll like receptor2/Nucleotide-Binding Domain, Leucine-Rich–Containing Family, Pyrin Domain–Containing-3/Caspace1/Interleukin-18 axis (TLR2/NLRP3/CASP1/IL-18) axis may promote IL18 mediated Treg upregulation in the early phase of infection. The resulting immune tolerance may be the basis for protection against allergy and possibly autoimmune disorders such as MS and MOGAD. On the other hand, chronic gastritis is characterized by upregulated T helper 17(Th17) response, neutrophil recruitment and expression of B cell activating factor (BAFF) in macrophages (35, 36). Chronic *Hp* infection has also been noted to be associated with gut dysbiosis and associated pro inflammatory state (37). We have postulated that pro- inflammatory state of chronic gastritis accompanied by gut dysbiosis may upregulate Th1/Th17 driven immune response initially in the gut and later systemically, facilitating the development of NMOSD in later life. The role of gastric derived antibodies that cross react with neural elements cannot be excluded. A previous publication from our group has shown this to be a potential pathway for disease pathogenesis in NMOSD (38). *Helicobacter pylori* related pro-inflammatory proteins particularly neutrophil activating protein (NAP) may contribute to AQP4 –IgG associated neural damage and severity of disease.In a recent study a significant correlation was noted between seropositive AQP4 IgG + NMOSD, *Hp*- NAP and disability (10). Bacterial virulence genes of *Hp* have not been studied in detail in the context of CNS autoimmune disorders. An association between *Hp*- cytotoxin associated gene A (cag-A) and autoimmune thyroiditis (39) and asthma was previously reported (28). Additionally the underlying genetic susceptibility for autoimmune disorders is also important.

We reviewed the relevance of hygiene hypothesis in a low middle income (LMIC) setting in relation to *Hp* infection and CNS autoimmune disorders. For this purpose,we analysed educational status and income levels of patients and primary caregivers in childhood in order to correlate poor sanitary conditions in childhood with early *Hp* colonization in the gut. Our study showed a significant association between lower educational status among all cases and controls stratified by *Hp* serology. Our hospital caters to patients from lower economic status which may explain the lack of difference in economic status between the two groups.

In conclusion, in developing countries *Hpylori* may be a significant environmental factor associated with autoimmune demyelinating disorders. Our study was limited by the small number of study participants and the cross sectional nature of the analysis. It is not clear at this time whether analysis of risk/benefit has to be carefully undertaken before *Hp* eradication is contemplated, in populations harbouring high *Hp* seroprevalence. Therefor the role of *H pylori* in modulating human immunity and its protective/deleterious effects need to be understood through larger studies that will additionally evaluate the role of *Hp* virulence genes.

## Data availability statement

The original contributions presented in the study are included in the article/**Supplementary Material**. Further inquiries can be directed to the corresponding author.

## Ethics statement

The studies involving human participants were reviewed and approved by Central Ethics Committe, Nitte University. The patients/participants provided their written informed consent to participate in this study.

## Author contributions

LP developed the concept, study design, analysis, interpretation, drafting and revising the work. CM contributed to study design, data acquisition, analysis, interpretation of results, manuscript drafting and revision. AD’C contributed to data acquisition, analysis and manuscript drafting. AS contributed to data collection, data analysis and interpretation. All authors contributed to the article and approved the submitted version.
