# Supplementary material for: Helicobacter pylori infection may influence prevalence and disease course in myelin oligodendrocyte glycoprotein antibody associated disorder (MOGAD) similar to MS but not AQP4-IgG associated NMOSD
Source: Front Immunol. 2023 May 26;14:1162248. doi: 10.3389/fimmu.2023.1162248 (PMC10250711; doi:10.3389/fimmu.2023.1162248)
Supplement: Supplementary file 1 [file Table_1.docx]

Supplementary Files

Table 1 - *H pylori* antibody frequency among subtypes of demyelinating disorders.

A, MS versus NMOSD

|  | MS Cases(n=254) | NMOSD (n=99) | P value |
| --- | --- | --- | --- |
| H pylori positive | 54 (21.2%) | 42 (42.4%) | **0.0006** |
| H pylori negative | 200(78.8%) | 57 (57.6%) |  |

B, MOGAD versus NMOSD

|  | MOGAD (n= 99) | NMOSD (n=99) | P value |
| --- | --- | --- | --- |
| H pylori positive | 28(28.3%) | 42 (42.4%) | **0.038** |
| H pylori negative | 71(71.7%) | 57 (57.6%) |  |

C, MS versus MOGAD

|  | MS Cases(n=254) | MOGAD (n =99) | P value |
| --- | --- | --- | --- |
| H pylori positive | 54 (21.2%) | 28(28.3%) | 0.16 |
| H pylori negative | 200(78.8%) | 71(71.7%) |  |

D, MS & MOGAD combined Versus NMOSD

|  | MS+MOGAD (n=353) | NMOSD (n=99) | P value |
| --- | --- | --- | --- |
| H pylori positive | 82(23.2%) | 42 (42.4%) | **0.0001** |
| H pylori negative | 271(76.8%) | 57 (57.6%) |  |

Table 2 MOGAD-MS :

*Hpylori*seropositive (n=82) versus seronegative(n=271)patients (Univariate analysis )

| Characteristics | *Hp+* | *Hp -* | P value | Odds ratio | 95% CI |
| --- | --- | --- | --- | --- | --- |
| Disease duration (Mean± SD) | 10.03±7.21 | 8.6±6.1 | 0.14 | 1.03 | 0.99-1.07 |
| Age at onset (Mean±SD) | 31.1±12.01 | 26.7±11.3 | **0.001** | 1.03 | 1.01-1.05 |
| EDSS (Mean±SD) | 2.29±2.61 | 2.39±2.37 | 0.21 | 1.04 | 0.94-1.15 |
| Gender  Male  Female | 34(41.4%)  48(58.6%) | 100(36.9%)  171(63.1%) | 0.45 | 1.21 | 0.73-2.01 |
| Education  Basic education  Higher education | 49(62.8%)  29(37.2%) | 117(45.9%)  138(54.1%) | **0.009** | 1.99 | 1.18-3.36 |
| Socioeconomic status  Low  High | 43(54.4%)  36(45.6%) | 112(44%)  142(56%) | 0.11 | 1.51 | 0.91-2.51 |
| Area of living  Rural  Urban | 39(50%)  39(50%) | 106(41.9%)  147(58.1%) | 0.21 | 1.39 | 0.83-2.31 |

Abbreviations: EDSS= Expanded disability status scale, *Hp+* = *Helicobacter pylori* positive, Hp - = *Helicobacter pylorinegative.*

Table 3:MOGAD-MS :

Multivariate analysis *H pylori* seropositive versus seronegative in MOGAD/MS patients

| Variables | p value | Odds ratio | 95%CI |
| --- | --- | --- | --- |
| Age at onset  Disease duration  EDSS  Socioeconomic status  Area of living  Education status | **0.001**  **0.04**  0.21  0.97  0.96  **0.02** | 1.04  1.04  0.92  1.01  0.98  2.12 | 1.01-1.06  1.002-1.08  0.82-1.04  0.51-1.96  0.52-1.87  1.11-4.06 |

Table 4. MS patients

**Univariate analysis of *H pylori* seropositive(n=54) versus seronegative(n=200) MS patients:**

| Characteristics | *Hp+* | *Hp-* | P value | OR | 95% CI |
| --- | --- | --- | --- | --- | --- |
| Disease duration (Mean±stdv) | 10.8±6.61 | 9.35±6.39 | 0.15 | 1.03 | 0.98-1.08 |
| Age at onset(Mean±stdv) | 32.3±12.4 | 27.4±9.8 | **0.003** | **1.04** | **1.01-1.07** |
| EDSS (Mean±stdv) | 2.8±2.2 | 2.69±2.5 | 0.35 | 1.01 | 0.89-1.15 |
| Gender  Male  Female | 17(31.5%)  37(68.5%) | 62(31%)  138(69%) | 0.9 | 1.02 | 0.53-1.95 |
| Education  Basic education  Higher education | 29(55.8%)  23(44.2%) | 75(38.5%)  120(61.5%) | **0.02** | **2.01** | **1.08-3.74** |
| Socioeconomic status  Low  High | 28(52.8%)  25(47.2%) | 74(37.9%)  121(62.1%) | **0.05** | **1.83** | **0.99-3.37** |
| Area of living  Rural  Urban | 26(49%)  27(51%) | 73(37.6%)  121(62.4%) | 0.13 | 1.59 | 0.86-2.94 |

Table 5: MS patients

**Multivariate analysis *H pylori* seropositive versus seronegative in MS patients**

| Variables | p value | Odds ratio | 95%CI |
| --- | --- | --- | --- |
| Age at onset  Disease duration  Socioeconomic status  Area of living  Education status | **0.008**  0.15  0.44  0.95  0.37 | 1.04  1.03  1.36  1.02  1.42 | 1.01-1.07  0.98-1.09  0.61-3.05  0.45-2.28  0.65-3.09 |

Table 6 : MOGAD patients

**Univariate analysis of *H pylori* seropositive (n=28) versus seronegative (n=71)MOGAD patients:**

| Characteristics | *Hp+* | *Hp-* | P value | OR | 95% CI |
| --- | --- | --- | --- | --- | --- |
| Disease duration (Mean±stdv) | 8.44±8.24 | 6.29±4.46 | 0.12 | 1.06 | 0.98-1.14 |
| Age at onset (Mean±stdv) | 28.7±11.1 | 24.9±14.9 | 0.2 | 1.01 | 0.98-1.05 |
| EDSS (Mean±stdv) | 2.24±2.68 | 1.44±1.62 | 0.11 | 1.19 | 0.96-1.48 |
| Gender  Male  Female | 17(60.7%)  11(39.3%) | 38(53.5%)  33(46.4%) | 0.51 | 1.34 | 0.55-3.27 |
| Education  Basic education  Higher education | 20(76.9%)  6(23.1%) | 42(70%)  18(30%) | 0.10 | 2.34 | 0.82-6.6 |
| Socioeconomic status  Low  High | 15(57.7%)  11(42.3%) | 38(64.4%)  21(35.6%) | 0.61 | 0.78 | 0.30-1.99 |
| Area of living  Rural  Urban | 13(52%)  12(48%) | 33(56%)  26(44%) | 0.9 | 0.96 | 0.38-2.40 |

Table 7: MOGAD patients

**Multivariate analysis *H pylori* seropositive versus seronegative in MOGAD patients**

| Variables | p value | Odds ratio | 95%CI |
| --- | --- | --- | --- |
| Age at onset  Disease duration  EDSS  Education status | 0.14  0.11  0.68  0.10 | 1.03  1.08  1.06  2.44 | 0.99-1.07  0.98-1.18  0.79-1.41  0.82-7.27 |

Table 8: NMOSD patients

Univariate analysis- *H pylori* seropositive versus seronegative in AQP4 IgG+ NMOSD patients:

| Characteristics | *Hp+* | *Hp -* | P value | Odds ratio | 95%CI |
| --- | --- | --- | --- | --- | --- |
| Disease duration (Mean± SD) | 9.97±7.48 | 11.27±7.18 | 0.34 | 0.97 | 0.91-1.04 |
| Age at onset (Mean±SD) | 31.56±12.53 | 31.55±13.05 | 0.93 | 1.00 | 0.96-1.03 |
| EDSS (Mean± SD) | 5.14±3.48 | 3.73±3.07 | 0.08 | 1.14 | 0.97-1.33 |
| BMI (Mean±SD) | 22.9±3.14 | 23.47±5.05 | 0.89 | 0.97 | 0.86-1.08 |
| Gender  Male  Female | 6 (14.2%)  36(85.8%) | 3(5.3%)  54(94.7%) | 0.12 | 3.00 | 0.70-12.77 |
| Education  Basic education  Graduation and above | 21(72.4%)  8(27.5%) | 26(42.2%)  19(57.8%) | 0.21 | 1.91 | 0.7-5.25 |
| Socioeconomic status  Low  High | 22(78.6%)  6(21.4%) | 22(48.9%)  23(51.1%) | **0.01** | **3.83** | **1.31-11.23** |
| Area of living  Rural  Urban | 19(79.2%)  5(20.8%) | 21(56.8%)  16(43.2%) | 0.07 | 2.89 | 0.88-9.42 |
| Diet  Veg  Non veg | 7(24.1%)  22(75.9%) | 15(33.3%)  30(66.7%) | 0.4 | 0.63 | 0.22-1.82 |

Table 9:

Multivariate analysis *H pylori* seropositive versus seronegative in NMOSD

| Variables | p value | Odds ratio | 95%CI |
| --- | --- | --- | --- |
| Gender  EDSS  Socioeconomic status  Area of living  Education status | 0.28  0.11  0.10  0.41  0.65 | 2.9  1.16  3.61  1.75  1.48 | 0.4-21.9  0.96-1.41  0.77-16.9  0.45-6.79  0.26-8.4 |
